# The Evolution of Consent Law in the UK

**DOI:** 10.1186/1749-8090-10-S1-A202

**Published:** 2015-12-16

**Authors:** Sparsh Prasher, Michael Klimatsidas, Zahid Mahmood

**Affiliations:** 1 Golden Jubilee National Hospital, Agamemnon St, Clydebank, Dunbartonshire G81 4DY, UK

## Background/Introduction

Every human has a fundamental right, to determine what happens to their body (autonomy). For us as surgeons, it is imperative that we obtain valid consent from our patients before performing any examination or procedure. For consent to be valid, it must be informed, voluntary, and be given by a competent patient.

There is an existing judicial debate in UK Law regarding how much information should be given to patients for the consent to be accepted as informed and therefore valid. Recently, the case of Montgomery vs NHS Lanarkshire has changed the consent law in the UK.

## Aims/Objectives

We aim to describe the evolution of consent law in the UK

## Method

A detailed literature search was performed in the British and Irish Legal Information Institute to examine case laws relating to consent

## Results

Landmark cases on informed consent in British Law have been provided in the table attached.

**Table 1:** Evolution of Consent Law in The UK-Important Case Laws

Case (Year)	Nature of Case	Ruling	Significance
Bolam vs Friern Hospital (1957)	The patient (Bolam) underwent electroconvulsive therapy for depression and suffered pelvic fracture. Bolam filed a negligence claim as he was not informed of the risk of injury.	The judgement was based if the doctor's practice was deemed acceptable by other competent doctors-This became known as the Bolam test. The doctor was not held liable his actions were supported by his peers.	Introduced the concept of the Bolam test.

Evolution of Consent Law in The UK- Important Case Laws Case (Year) Nature of Case Ruling Significance Bolam vs Friern Hospital (1957) The patient (Bolam) underwent electroconvulsive therapy for depression and suffered pelvic fracture. Bolam filed a negligence claim as he was not informed of the risk of injury. The judgement was based if the doctor's practice was deemed acceptable by other competent doctors-This became known as the Bolam test.

The doctor was not held liable his actions were supported by his peers. Introduced the concept of the Bolam test.

## Discussion/Conclusion

The law has evolved by moving away from the paradigm of paternalism to emphasize the significance of true informed consent. Therefore, in modern surgical practice a patient must be given enough information in order to make a well thought out decision. This information includes the reasons for having surgery, what the procedure will involve, and the benefits and risks of the procedure. All risks that an average patient would want to know should be mentioned to the patient, even if the risk itself is very small. The effect of not having the proposed procedure should also be explained. It is also important to address the effect of the surgery on the quality of life of the patient as well as any effect on their religious and spiritual wellbeing (for example blood transfusion in Jehovah's witness). Therefore in contemporary surgical practice, especially in the authors practice, the term seeking consent can be replaced by the more progressive term- joint decision making. This term is more accurate as it highlights the mutual agreement between us as health professionals and our patients based on our clinical knowledge and expertise which then takes into consideration the patient's rights, values and choices.

